# The Significance and Limitations of Pre- and Postnatal Imaging in the Diagnosis and Management of Proximal Focal Femoral Deficiency

**DOI:** 10.3390/diagnostics15111302

**Published:** 2025-05-22

**Authors:** Aaron C. Llanes, Emma Venard, Sean Youn, Dane Van Tassel, Luis F. Goncalves, Mohan V. Belthur

**Affiliations:** 1 College of Medicine, University of Arizona, Phoenix, AZ 85016, USA; llanesa10@gmail.com (A.C.L.); emmasvenard@gmail.com (E.V.); sean.youn@bannerhealth.com (S.Y.); 2 Department of Radiology, Phoenix Children’s Hospital, Phoenix, AZ 85016, USA; dvantassel1@phoenixchildrens.com (D.V.T.); lgoncalves@phoenixchildrens.com (L.F.G.); 3 Department of Orthopedics, Phoenix Children’s Hospital, Phoenix, AZ 85016, USA

**Keywords:** PFFD, CFD, ultrasound, MRI, pediatric orthopedics

## Abstract

**Background and Clinical Significance**: Proximal femoral focal deficiency (PFFD), also referred to as congenital femoral deficiency, is a longitudinal limb deficiency and birth defect that affects the lower extremity including the hip and femur, resulting in a deformed and shortened limb. It can be diagnosed and classified using a combination of imaging modalities, including radiographs, ultrasonography, magnetic resonance imaging and computerized tomography. It is crucial to characterize this birth defect in the prenatal period to appropriately prepare parents through counseling. Postnatal imaging should be performed to confirm the diagnosis, prognosticate and predict the patient’s course for treatment and management. Close follow-up and family/patient-centered care contribute to optimized patient outcomes. **Case Presentation**: Here, we present a series of three cases of varying PFFD severity and presentation, detailing the evaluation process, the limitations and value of imaging, and the treatment outcomes of these patients. Each case has a different PFFD classification and treatment strategy that we utilized according to the data that we attained through continuous patient care and discussion. **Conclusions:** We highlight the difficulties in identifying and classifying PFFD in the prenatal period while demonstrating how postnatal imaging clarified the diagnosis and informed appropriate counseling and treatment. Close follow-up and the length of patient continuity allowed us to maximize patient outcomes despite the variety in PFFD presentation and severity.

## 1. Introduction

Proximal femoral focal deficiency (PFFD), also referred to as congenital femoral deficiency (CFD), is a birth defect that affects the lower extremity including the hip and femur, resulting in a shortened limb [1]. PFFD is exceedingly rare, presenting in 1.1–2.0 out of 100,000 live births [2,3]. While the cause of PFFD is unclear, some studies have associated PFFD with known teratogens such as thalidomide, as well as risk factors including hypoxia, ischemia, and diabetes mellitus within the maternal womb [4,5]. PFFD may present as either bilateral or unilateral limb deficiency, and the severity of PFFD is categorized based on the degree of lower extremity involvement [6,7,8,9,10].

PFFD evaluation is critical in predicting patient outcomes and for establishing the appropriate intervention and management strategy, for which multiple PFFD classification systems have been developed, each with their own idiosyncrasies. For instance, the Aitken system categorizes PFFD into a spectrum of severity from A to D dependent upon the presence or absence of the acetabulum, femoral head, femoral segment, and attachment of femoral head to shaft [6]. Left untreated, PFFD can prove to be highly debilitating to the patient, both in terms of physical disability and psychosocial impediment [11,12]. The management of PFFD is dependent on the goals of the patient and can range from prosthetic reconstruction with orthoses and prostheses to limb reconstruction with surgical intervention to stabilize, realign, and lengthen the affected limb or limbs [12,13,14,15,16,17,18]. Here, we present a series of three cases of varying severities encountered at a tertiary children’s hospital. We provide insight into the challenges we faced in the evaluation process, during patient and family discussions regarding treatment strategy, and in patient continuity to achieve positive outcomes.

## 2. Case Series

### 2.1. Case 1

This female patient was born to a G2P1 mother, who refused permission for the inclusion of images. The mother was first seen at 26 weeks’ gestation after a 20-week anatomy scan showed a shortened right femur and absent tibia. The pregnancy was complicated by type II diabetes mellitus managed with insulin, maternal right bundle branch block, hypothyroidism, hypertension, and obesity.

Ultrasonography (US) of the fetal right lower extremity identified a punctate echogenic focus adjacent to the pelvis and a single bowed distal osseous structure that articulated with an abnormally rotated foot. A parasagittal balanced turbo field echo MRI sequence helped to elucidate the corresponding focus as a dysmorphic and shortened femur; however, the identity of the distal osseous structure as either tibia or fibula was indeterminate. Its measurement at 1.68 cm at 26 weeks of gestational age was markedly below the mean length for either bone, registering approximately 11 standard deviations below the mean for tibial length and 10.4 standard deviations for fibular length, nonetheless consistent with profound shortening [19]. Classification for the right lower extremity at the time was Aitken type D due to the diminutive hypoechogenic proximal structure and distal single long bone [6]. The left lower extremity showed a short, curved femur and subjectively decreased muscle bulk of the left calf thought to classify Aitken type B. The patient was counseled by Orthopedic Surgery with the plan of performing a comprehensive clinical and imaging evaluation after birth. The pregnancy and the delivery management were not altered as a result of the prenatal diagnosis.

A 3465 g newborn was delivered at 39 weeks and 2 days via cesarean section. The right lower extremity was hypoplastic, and the left lower extremity had severe clubbing at birth. Radiographs of the pelvis and hips performed shortly after birth revealed a dysplastic left hip, a left femur measuring 55.9 mm, a normal left fibula measuring 57.8 mm, a normal left tibia measuring 60.3 mm, and left clubfoot. There was anterolateral bowing of the left femur, which was foreshortened compared with the left tibia and fibula. The left leg length from the ossification center of the proximal left femoral epiphysis to the tibial plafond measured 124.8 mm, though the curvature of the left femur made underestimation likely. The right hip was severely dysplastic with an amorphous faint calcification in the expected area of the right femur, and a single distal right leg bone with a rounded, well-developed proximal ossification center. This bone, later confirmed to be the fibula by MRI, was medially bowed and gracile in appearance compared with the left. A dislocated right ankle and bilateral talipes equinovarus were also seen. These findings were consistent with bilateral PFFD, Aitken class D for the right lower extremity and Aitken class B for the left lower extremity.

Bilateral MRI of the hip without contrast clarified that the right tibia was absent, and the distal right long bone was more suggestive of a fibula. The scan also revealed fibrous change in the region of the expected right superior pubic rami, a small cartilaginous area likely representative of the right distal femoral condyle, and no sign of articulation between the distal right lower extremity long bone and right foot. The left femur was shown to have a bowing deformity, but the proximal cartilaginous portion of the femur appeared grossly normal and related appropriately to the triradiate cartilage/acetabulum of the left hemipelvis with an appropriate left knee joint and suggested articulation of the left distal tibia and left talus. Musculature was significantly decreased on the right and distally decreased on the left.

The patient was first seen by general pediatrics at 17 days old; at which point, a physical exam showed a significantly shorter right leg without a clear tibia or fibula and left lower extremity clubbing with decreased tone in lower extremities bilaterally. The plan at the time was to follow-up with orthopedics and genetics.

Genetic evaluation attributed this case of PFFD to teratogenic causes given the diabetic status of the mother. Follow-up evaluation by orthopedics resulted in a diagnosis of bilateral PFFD, more severe on the right side, right intercalary deficiency of tibia and fibula, bilateral hip abduction and flexion contractures, bilateral knee flexion contractures, and bilateral syndromic clubfeet. As of today, the patient has not undergone any surgical repair although prosthetics are currently under consideration. The patient is now 4 years old and developing well despite some restrictions. She mobilizes by scooting on her bottom at home and uses a gait trainer for short distances and a wheelchair for longer distances.

### 2.2. Case 2

**Figure 1:**
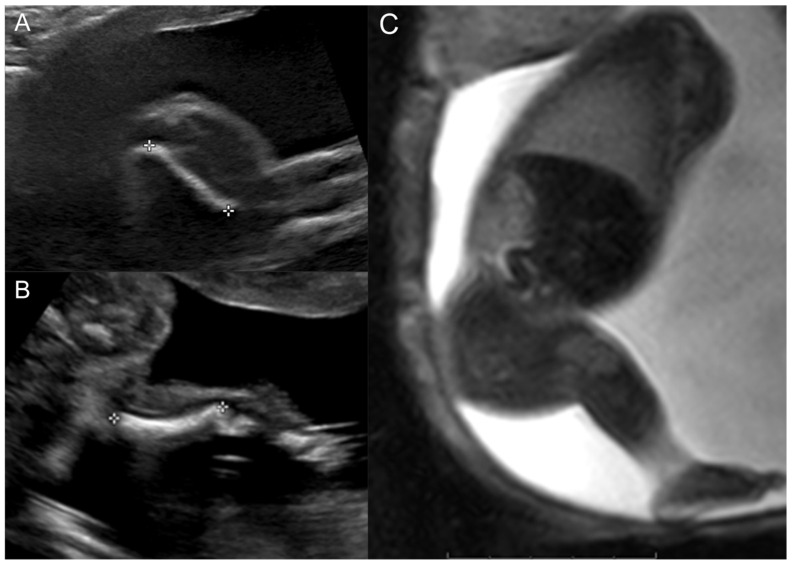
Prenatal right lower extremity examination at 23 weeks gestation. (**A**) Right lower extremity US demonstrating short femur. (**B**) Right lower extremity US demonstrating the presence of a single bone in the leg (tibia) and absent fibula. (**C**) Thick slab balanced turbo field echo MRI images of the entire right lower extremity.

The female patient was born to a G4P3 mother. The mother was seen for a fetal US and MRI at 23 weeks gestation. At the time, the fetus was known to have a short right femur with absent tibia and missing vs. hypoplastic right fifth metatarsal and toe. US showed a shortened right femur of 2.0 cm vs. 4.5 cm on the left, a short right tibia of 2.6 cm vs. 3.5 cm on the left, and an absent distal right fibula vs. a 3.8 cm left fibula (Figure 1). The left foot showed no abnormalities, whereas the right foot appeared to have a missing fifth digit. The findings were consistent with right PFFD and ipsilateral fibular hemimelia. Similarly, fetal MRI showed a short right femur, rudimentary right distal fibula, and possible longitudinal ray defect of the fifth digit of the right foot.

The patient was born at 36 weeks with a birth weight of 2948 g and was first seen at the age of 8 months. Physical exam revealed a globally shortened right lower extremity with a positive Galeazzi test, shortened right tibia with mild anterior medial bowing, no palpable fibula, a right four-ray foot, a right knee with full flexion and 10–15 degrees short of full extension, a 20–30 degree right hip flexion contracture, and a right foot at the level of the proximal third of the left tibia during right leg extension. Further examination revealed preserved right subtalar range of motion and mild right genu valgum.

**Figure 2:**
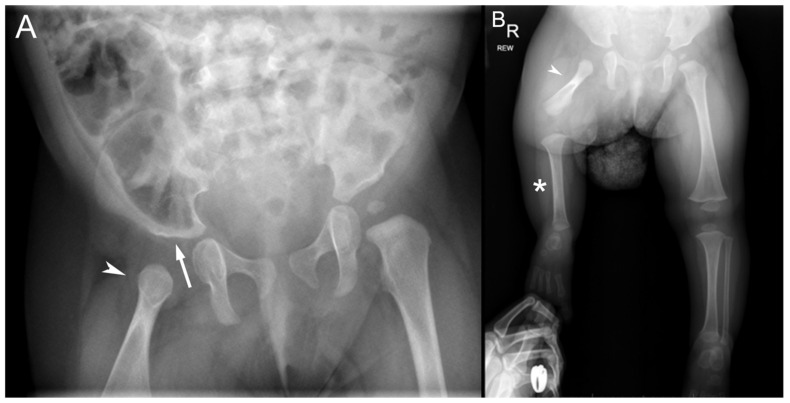
Radiographs at 8 months of age. (**A**) Pelvis radiograph shows a dysplastic right acetabulum (white arrow) and malformed proximal right femur (arrowhead). The proximal epiphysis, metaphysis, and lesser trochanter are unossified. (**B**) Bilateral lower extremity radiograph shows the hypoplastic right femur (arrowhead) consistent with PFFD, absent right fibula (*) consistent with fibular hemimelia. The contralateral left femur, tibial, and fibula are normal.

Bone length studies at the time showed a length of 6.7 cm between the right proximal femur (most proximal ossified portion of the right femur) and the right medial femoral condyle, 11.6 cm between the right medial femoral condyle and tibial plafond, and right leg length of 18.3 cm. In comparison, the left femoral head to medial femoral condyle measured 15.6 cm; the left medial femoral condyle to tibial plafond measured 13.1 cm; and the left leg length measured 28.7 cm. A bilateral hip with pelvis radiograph showed a well-located left hip with an acetabular index (AI) of 21 degrees, femoral head ossification, and intact Shenton’s arc. The right femoral head was unossified on anteroposterior (AP) radiographs with an apparent reduction on a lateral frog leg radiograph (Figure 2A). The long leg radiograph showed complete right fibular absence and a right femur of 66 mm compared with a left femur of 139 mm. The right tibia measured 92 mm, while the left tibia measured 112 mm. The right foot showed four fully formed rays with a normal-appearing calcaneus and talus (Figure 2B). Overall, the right leg was 11.4 cms shorter than the left with a projected leg length discrepancy at maturity of 40 cms. The patient was diagnosed with a right fibular hemimelia and a right PFFD. After further evaluation, the plan was for prosthetic reconstruction surgery with an interim right lower extremity extension prosthesis at age 2 years and a Van Nes rotationplasty at age 4 years followed by a below-knee prosthesis.

At age 2 years, the patient was able to ambulate and crawl using her left knee and was not using or wearing her right lower extremity extension prosthesis. However, by age 3 years and 10 months, the patient was falling a lot while walking after outgrowing and discontinuing her prosthesis and compensated by crawling. Limb length discrepancy was 12 cm at this time, with the right femur measuring ~50% of the left and the right tibia measuring ~66% of the left. Hip flexion contracture and knee flexion contracture were 15 degrees and 5 degrees, respectively. Ankle valgus was seen on standing, and the ankle equinus measured 20 degrees. The range of motion was well preserved in the hips, knees, ankles, and feet bilaterally. The patient was classified as Gillespie–Torode functional group 1B at this time [20]. Bilateral lower extremity bone length radiographs at this appointment showed a left lower extremity of 45.7 cm compared with a right lower extremity of 30.0 cm, along with right coxa vara, right femoral retroversion, right genu valgum, and right ankle valgus. The plan at this time was for a right SUPERhip (Systemic Utilitarian Procedures for Extremity Reconstruction) procedure in preparation for a Syme amputation and follow-up with Prosthetics for a transtibial prosthesis.

Bone length studies 3 months prior to the surgery showed a right proximal femur to medial femoral condyle length of 13.4 cm, right medial femoral condyle to tibial plafond of 16.9 cm, and right leg length of 30.3 cm compared with a left femoral head to medial femoral condyle length of 25.2 cm, left medial femoral condyle to tibial plafond length of 20.2 cm, and left leg length of 45.4 cm with unchanged absent right fibula, deformed proximal right femur, and absent right fifth ray.

The patient underwent the SUPERhip procedure including right proximal femoral corrective osteotomy with internal fixation, right pelvic Dega osteotomy, right iliopsoas tenotomy over the brim, right rectus femoris release and transfer to sartorius, right femoral nerve decompression, right distal iliotibial band excision, transfer of the tensor fascia lata to the greater trochanter, and right distal femoral medial hemiepiphysiodesis with placement in 1.5 spica cast at age 4 years and 2 months [21]. The patient was referred to physical therapy and continued to follow-up with prosthetics.

**Figure 3:**
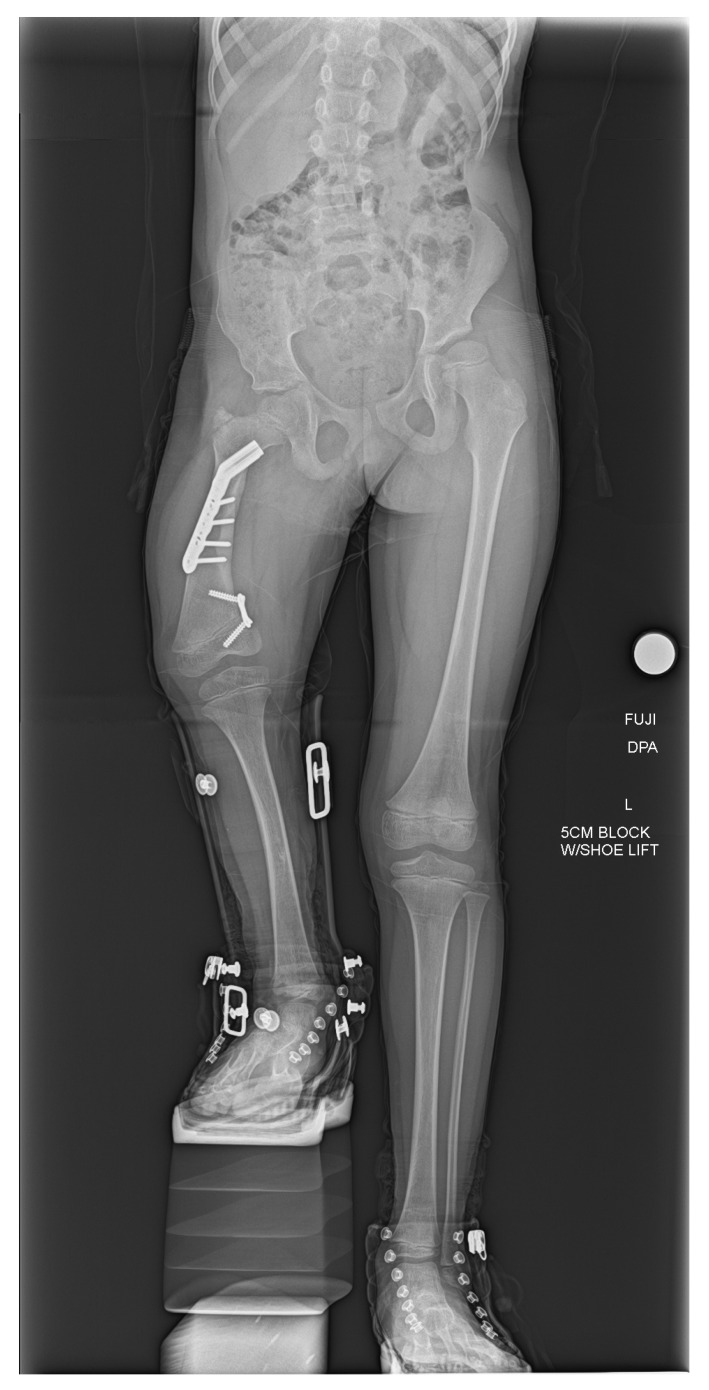
AP bilateral lower extremity radiograph post-SUPERhip procedure. Right femoral osteotomies are healing appropriately, and hardware is intact and well tolerated.

At the last follow-up visit, the patient was doing well and walking without issues with a right extension prosthesis. The most recent radiographs of the right femur showed healing osteotomies in the right proximal femur and the pelvis well-positioned with retained hardware (Figure 3). The current plan is to follow-up with physical therapy, and with the prosthetist for a new prosthesis, as well as with orthopedics for follow-up evaluation and right femur radiographs.

### 2.3. Case 3

**Figure 4:**
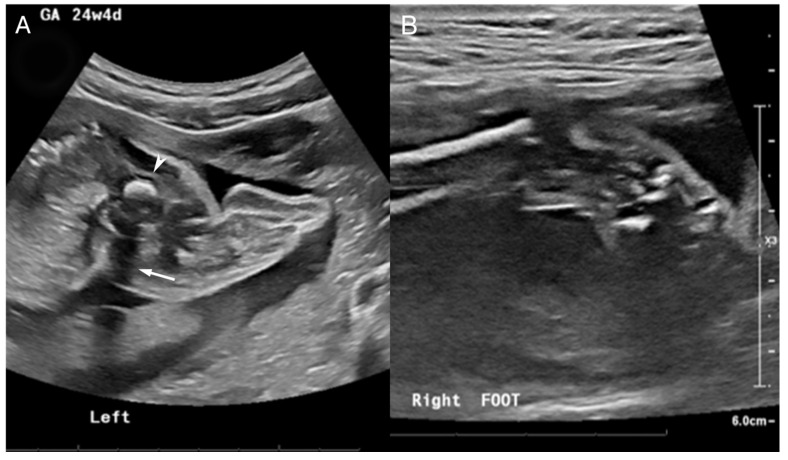
US at 24 4/7 weeks gestation. (**A**) Abnormal left lower extremity US noted by a hyperechogenic focus (arrowhead) with posterior shadowing (white arrow) corresponding to a diminutive femoral remnant adjacent to the pelvis. No bones are identified in the left calf and foot. (**B**) Right ankle US shows a partially visualized clubfoot.

The patient was born to a G2P1 mother. The mother was first seen at 24 weeks gestation after a 20-week fetal US showed a small dysplastic left femoral head with abnormal cartilage and lack of visualization of the left tibia and fibula osseous structures (Figure 4A). Questionable cartilaginous structures were seen on follow-up MRI. The left calf soft tissue and musculature were visualized to be partially formed at this time. The left foot was shown to articulate with the partially formed and shortened left tibia and fibula. Hindfoot equinus and hindfoot varus plus forefoot varus were seen on the right (Figure 4B). The patient was diagnosed with a likely left PFFD with an abnormally bowed and shortened left tibia and fibula.

The patient was born at 35 weeks at 2664 g via cesarean section. A Physical exam at this time showed well-preserved right hip and knee range of motion with right ankle equinus −20 and right idiopathic typical clubfoot with a Pirani score of 5/6 with a hindfoot score (HFS) of 3/3 and a midfoot score (MFS) 2/3 along with an overriding of the fourth toe on the third [22]. The left leg exam revealed a hypoplastic left leg with a markedly short femur, absent tibia and fibula, a three-ray foot, and a valgus ankle with the joint above the level of the contralateral knee joint. There was significant hip abduction, external rotation, and flexion contracture. The findings were significant for Aitken type D PFFD. The final diagnosis was severe proximal femoral deficiency and fibular hemimelia. The patient was functionally labeled as Gillespie–Torode type C as the left ankle was above the level of the right knee, with a predicted final leg length discrepancy >40 cm [20].

**Figure 5:**
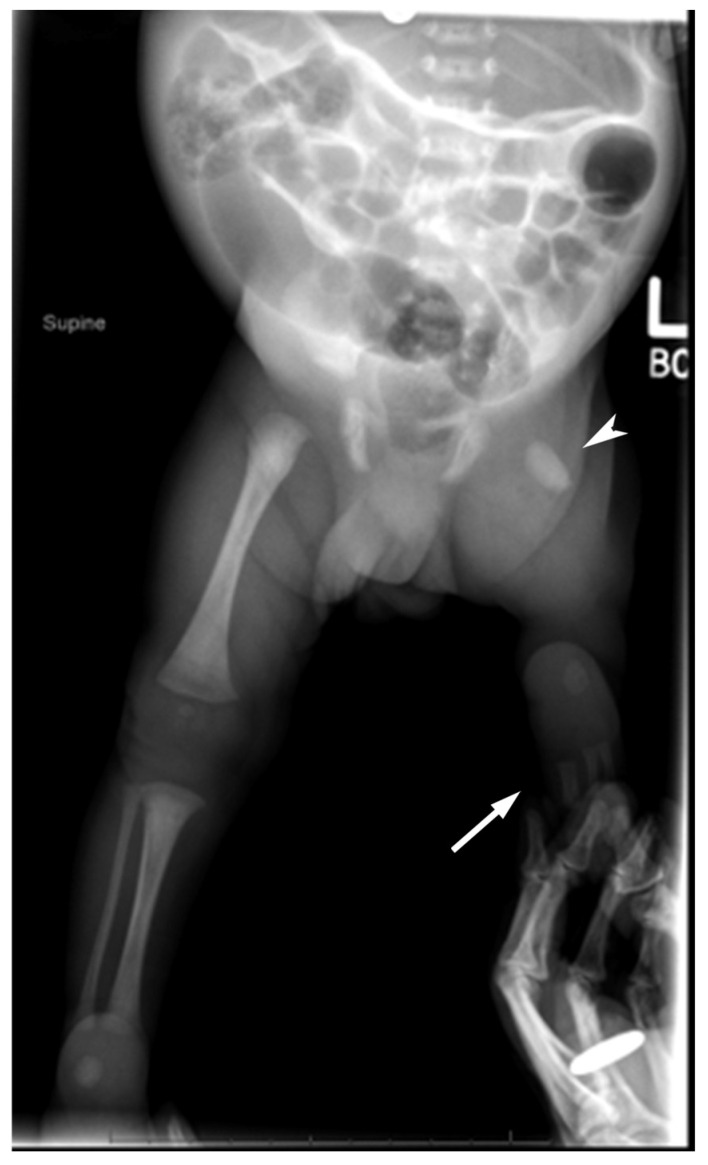
AP bilateral lower extremity radiograph at 6 months of age shows a diminutive left femur (arrowhead), no ossification of the tibia or fibula (white arrow), and 3 digital rays in the left foot.

A radiograph bone length study shortly after birth showed a right femur length of 7.5 cm and right tibia length of 6.3 cm from ossified metaphysis to metaphysis. The left lower extremity was unable to be measured due to the patient’s PFFD. Follow-up radiograph bone length at age 6 months revealed a normal-appearing right lower extremity and deficiency of the left lower extremity with proximal ossification of the femoral head and neck without ossification of the rest of the femur or the distal lower extremity with the exception of the left foot (Figure 5).

**Figure 6:**
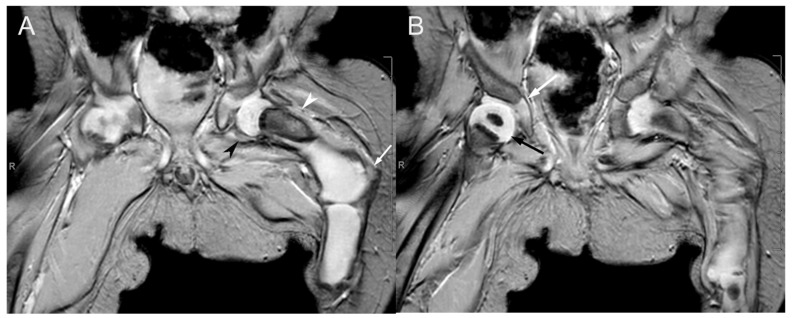
Coronal T2*-weighted MRI sequences of the proximal femora at one year of age. (**A**) The cartilaginous proximal left femoral epiphysis articulates with the left acetabulum. Only an oval-shaped ossified portion of the left femur is visible at the proximal femoral neck (hypointense region indicated by the white arrowhead). The remaining portions of the left femur are cartilaginous (hyperintense region indicated by the black arrowhead), with lateral bowing at the transition between the left femoral neck and the proximal diaphysis (white arrow). (**B**) The partially visualized right femur demonstrates normal articulation of the proximal femoral epiphysis (black arrow) with the right acetabulum (white arrow) and normal ossification of the proximal right femoral diaphysis.

At 15 months, a repeat radiograph bone length showed a length of 15.1 cm from the right femoral head to the medial femoral condyle, 14.4 cm from the right medial femoral condyle to the tibial plafond, and a right leg length of 29.5 cm. The left leg showed an overall measurement of 10.1 cm from the top of femur to the ankle/talus. The leg length discrepancy was noted to be around 19.4 cms with a predicted leg length discrepancy of greater than 40 cms. An MRI of the left hip and thigh without contrast at the time revealed a short segment of an ossified left femoral neck and proximal diaphysis with the remaining left femur appearing cartilaginous, significantly shortened, and deformed. A linear oblique low T2 signal abnormality was seen at the osseous–cartilaginous junction of the left femur with mild lateral bowing. A cartilaginous, shortened, dysmorphic tubular structure likely representing the tibia was seen articulating with the distal cartilaginous left femur. No left fibula was clearly visualized (Figure 6). The left calf, ankle, and foot were also significantly dysmorphic, and the muscles of the left lower extremity were atrophied.

The plan at the time was Ponseti treatment for the right clubfoot with an initial casting phase followed by a percutaneous tendoachilles tenotomy and a secondary bracing phase with a full-time brace for 4 months and part-time bracing up to 4 years [23]. The plan for the overriding right toes was to wait to address if they became an issue for wearing shoes later in life. The plan for the left lower extremity was to refer to prosthetics to discuss eventual prosthetic reconstruction surgery and management of the limb deficiency. Given the large leg length discrepancy, the patient was not considered for lengthening reconstruction surgery. The patient began the Ponseti treatment as instructed, as well as right percutaneous tendoachilles tenotomy and right long-leg Ponseti cast application at age 2 months.

**Figure 7:**
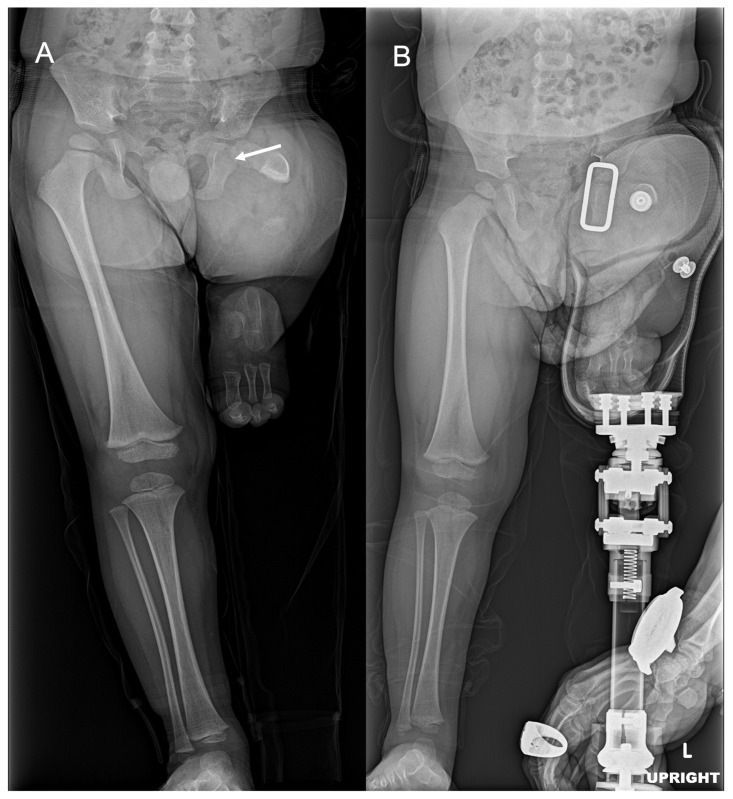
AP bilateral lower extremity radiographs with and without prosthesis. (**A**) Short and ossified portion of the left femoral neck and proximal diaphysis (white arrow). (**B**) Standing with left transfemoral prosthetic.

Today, the patient is a K2 ambulator and is expected to achieve and maintain K3 (Community ambulator)-level ambulatory status with a well-fitted abduction–dorsiflexion mechanism [24]. The patient attends physical therapy twice a week, is able to stand without the prosthesis, and is close to independent walking with a left transfemoral prosthesis. The patient continues to wear his braces part time during nights and naps as directed, and his right clubfoot is currently well corrected. The most recent radiographs show a good location of the left hip, ossification of the left proximal femur, and an ossified femoral head without acetabular dysplasia. The radiographs also demonstrated a short and ossified portion of the left femoral neck and proximal diaphysis that was directed toward the acetabulum (Figure 7). The bone length study for this patient showed a length of 19.2 cm from the right femoral head to the medial femoral condyle, a length of 15.6 cm from the right medial femoral condyle to the tibial plafond, and a right leg length of 34.7 cm with redemonstration of left femur deficiency and left tibia and fibula hemimelia and a left leg length of 12 cms with a predicted left limb discrepancy at skeletal maturity of greater than 40 cm at this time. The current plan is for continued physical and occupational therapy and close follow-up in the multidisciplinary prosthetic reconstruction clinic with a transfemoral prosthesis socket.

## 3. Discussion

Here, we present three cases to highlight the phenotypic variety in PFFD and presentation, the limitations and importance of prenatal and postnatal imaging as well as close follow-up in guiding diagnosis, patient counseling, and management of PFFD. PFFD can complicate growth and development in patients as presentation varies from a noticeable truncation of the femur to complete absence of the femur and acetabulum [25]. It may also present in isolation or with associated anomalies such as skeletal anomalies of the tibia, fibula, foot, arms and hands, as well as non-skeletal anomalies including vascular irregularities, ligament deficiencies, ventriculomegaly and oligoamnios, all of which should be factored into management goals, treatment, and overall patient outcomes [26,27,28,29,30,31].

Because of the phenotypic variety, multiple classification systems have been developed to categorize and characterize the severity of lower limb deformity and hypoplasia in PFFD. The most recognized and utilized classification systems include the Aitken (Table 1) and the Gillespie–Torode (Table 2) systems. Other systems include the Amstutz, Pappas, and Paley systems [32,33,34]. Each system has its own idiosyncrasies regarding anatomical descriptions and recommended treatment strategies [35]. In our cases, we used the Aitken system as the basis for classifying PFFD due to its simplicity in application using the femoral head and acetabulum as anatomical landmarks [6]. On follow-up, we further defined the degree of PFFD based on patient functionality using the Gillespie–Torode system due to its utility in recommending appropriate treatment and its parallels to the Aitken System [20]. Gillespie–Torode Class A is considered a stable congenital short femur; Gillespie–Torode Class B corresponds with Aitken classes A, B, and C; and Gillespie–Torode Class C corresponds with Aitken Class D [3,36].

Early diagnosis, treatment planning, and prognosis of PFFD are heavily influenced by prenatal evaluation. Fetal sonography plays a primary role in a preliminary diagnosis of PFFD as well as initial measurements for leg length discrepancies [29]. Indeed, the guidelines from the American Institute of Ultrasound in Medicine (AIUM) recommend ultrasound views of all extremities within the second trimester of pregnancy to screen for fetal deformities such as PFFD [37,38]. However, it is important to acknowledge the limitations of fetal ultrasonography. In our cases, PFFD was difficult to classify prenatally solely on ultrasound and required either supplemental MRI prenatally or postnatal confirmation with imaging to properly classify the defect. Indeed, while it may be desirable to measure each lower extremity bone, such as the femur, to better classify the extent of the defect during screening, factors such as the age of presentation and fetal position may make this impractical [38]. Additionally, previous works have noted that PFFD may be missed on sonographic screening due to subtlety or user experience [29].

By no means is this meant to insinuate futility or lack of ability but rather to highlight that prenatal imaging is only one aspect in PFFD evaluation as a whole. Despite these challenges, modern advancements in imaging modalities may make prenatal imaging more accurate. For instance, while ultrasonography has been employed to detect PFFD typically between 18 and 24 weeks of gestation [39], advances in ultrasonography have led to even earlier diagnoses of PFFD in the first trimester [40]. Kudla et al. report a case of PFFD diagnosed at 12 weeks of gestation using 3D and 4D ultrasound techniques [40].

Early detection of limb anomalies not only provides significant prognostic value for the affected patient but also anticipatory value for parents. Radler et al.’s survey demonstrated that 63% of mothers who gave birth to babies with PFFD would have preferred to learn the diagnosis before birth rather than after birth, highlighting the importance of prenatal evaluation in parental counseling and initiation of a management plan [41]. Therefore, future directions may look into advancements in prenatal ultrasound technology or MRI to more clearly define or sooner identify and classify the limb anomaly.

The postnatal evaluation of PFFD is a crucial component in establishing the continuity of care for patients with PFFD, especially as they grow and mature. As in our cases, MRI can be employed postnatally to clarify the extent of the deficiency and is especially helpful in classification [42]. However, MRI may not always be an option, possibly due to availability, cost, or other additional factors such as acquisition difficulty. CT scans can also be utilized, particularly when the acetabulum and proximal femur have ossified at an older age, although radiation exposure should also be considered [4]. An appropriate physical exam and follow-up radiographs, however, are standard in postnatal evaluation and onward to monitor the patient’s status and update the PFFD classification [43]. The data gathered from these exams are then used to track the trajectory of the limb’s development and guide the appropriate treatment.

A variety of treatments are available to manage PFFD, influenced by the severity of the malformation, associated limb deficiencies in other extremities, stability of the hip and knee, presence of joint contractures, and the family/patient’s goals such as comfort, functionality, and overall quality of life [10,12,18,44]. These strategies often amount to limb reconstruction surgery or prosthetic reconstruction surgery. Limb reconstruction may involve limb lengthening, arthrodesis, rotationplasty, amputation, or any combination of these strategies [10,12,21,45]. More specifically, surgical techniques such as Syme amputation of the foot, proximal or distal femoral osteotomy, innominate osteotomy, iliofemoral fusion, knee fusion, and Van Nes rotationplasty have been developed to facilitate the affected limb [11,20,35,46]. Prostheses can be developed and tailored to the patient or in conjunction with other interventions, like those previously mentioned [44,45]. However, close observation, as with our first case, is also a possibility until these options become viable on further discussion with the patient and family. Our cases with varying PFFD severity and treatments highlight the importance of personalizing patient management, despite the use of classification systems, to best fit the needs of the patient.

The outcomes of PFFD treatment may also vary depending on the chosen treatment. Unfortunately, limb lengthening procedures are commonly complicated by fractures of the femur once hardware is removed. While these fractures can be treated with techniques such as femoral nailing, this can also hamper the recovery of patients with PFFD [16]. Although rare, excessive limb lengthening may also be complicated by hip dislocation [47]. Additional complications of PFFD treatments reported in the literature include knee stiffness and decreased range of motion, joint subluxation, bone deformation, neovascular complications, and infection [14,15]. These complications can be prevented or managed through careful follow-up with the patient.

Despite the risks, patients with PFFD who have undergone intervention have demonstrated long-term success on follow-up [17,18,46]. In most successful cases of limb lengthening procedures, lengthening of up to 20–25% cm of the affected femur has been reported in a single treatment [14,15]. Studies by Ackman et al. and Kowalczyk and Kuznik-Buziewicz reported high functionality and quality of life for PFFD patients who had undergone Van Nes rotationplasty [17,46]. Westberry et al. report similar findings of functional ambulation in PFFD patients treated with knee arthrodesis [18]. However, a more recent study by Floccari et al. concluded that rotationplasty provided no patient-reported or functional benefit over prosthetic management [45]. In our cases, we owe our successful outcomes to close follow-up and continued conversations with the patients and their families.

## 4. Conclusions

PFFD is a rare, congenital lower limb anomaly with varying phenotypic presentations. Multiple classifications exist with which to describe the severity of the malformation as well as the feasible treatment options. A variety of imaging modalities are employed during prenatal, postnatal, and developmental evaluation to establish the diagnosis and classification. These data and discussions with the patient and family should guide appropriate treatment, which can vary from close observation to surgical intervention. Here, we exhibit three cases of PFFD that we encountered, each of varied severity. We detail the challenges we faced and describe the utility of prenatal and postnatal evaluation to classify the extent of malformation. We also describe the importance of follow-up and family discussion in guiding appropriate management and optimizing outcomes. We offer our insights into moderating expectations in prenatal evaluation, call for future advances in imaging that will improve the accuracy and congruence between pre- and postnatal imaging, and stress continued care to maximize patient outcomes.

## Figures and Tables

**Table 1:** Aitken classification.

Aitken Class	Femoral Head Morphology	Acetabulum Morphology
A	Present and attached to shaft	Normal
B	Present but no osseous connection seen between femoral head and shaft at maturity	Moderately dysplastic but contains head
C	Absent or abnormally small and not attached to femoral shaft	Severely dysplastic
D	Absent without an ossified tuft capping the proximal femur	Absent

**Table 2:** Gillespie–Torode classification.

Gillespie–Torode Class	Femoral and Knee Morphology	Treatment Strategy
A	Femur length >50% of unaffected femur Clinically stable hips No significant knee flexion contractures Ipsilateral foot at or below the middle of contralateral tibia	Limb lengthening
B	Femur length <50% of unaffected femur Foot at or above the level of the knee	Arthrodesis Rotationplasty Amputation Osteotomy Prosthesis
C	Subtotal absence of femur	Rotationplasty Amputation Osteotomy Prosthesis

## Data Availability

Supporting data is stored in a HIPPA protected file at Phoenix Children’s Hospital
